# Interlayer Affected Diamond Electrochemistry

**DOI:** 10.1002/smtd.202301774

**Published:** 2024-06-14

**Authors:** Xinyue Chen, Ximan Dong, Chuyan Zhang, Meng Zhu, Essraa Ahmed, Giridharan Krishnamurthy, Rozita Rouzbahani, Paulius Pobedinskas, Nicolas Gauquelin, Daen Jannis, Kawaljit Kaur, Aly Mohamed Elsayed Hafez, Felix Thiel, Rainer Bornemann, Carsten Engelhard, Holger Schönherr, Johan Verbeeck, Ken Haenen, Xin Jiang, Nianjun Yang

**Affiliations:** ^1^ Institute of Materials Engineering University of Siegen 57076 Siegen Germany; ^2^ Institute for Materials Research (IMO) Institute for Materials Research in MicroElectronics (IMOMEC) IMEC vzw Hasselt University Diepenbeek 3590 Belgium; ^3^ Electron Microscopy for Materials Research (EMAT) University of Antwerp Antwerp 2020 Belgium; ^4^ Physical Chemistry I Department of Chemistry and Biology and Department of Chemistry and Biology and Research Center of Micro and Nanochemistry and (Bio)Technology (Cµ) University of Siegen 57075 Siegen Germany; ^5^ Analytical Chemistry Department of Chemistry and Biology and Research Center of Micro and Nanochemistry and (Bio)Technology (Cµ) University of Siegen 57075 Siegen Germany; ^6^ Institute for High Frequency and Quantum Electronics University of Siegen 57076 Siegen Germany; ^7^ Department of Chemistry Institute for Materials Research in MicroElectronics (IMOMEC) IMEC vzw Hasselt University Diepenbeek 3590 Belgium

**Keywords:** doped diamond films, electrochemistry, interlayer, redox probes

## Abstract

Diamond electrochemistry is primarily influenced by quantities of sp^3^‐carbon, surface terminations, and crystalline structure. In this work, a new dimension is introduced by investigating the effect of using substrate‐interlayers for diamond growth. Boron and nitrogen co‐doped nanocrystalline diamond (BNDD) films are grown on Si substrate without and with Ti and Ta as interlayers, named BNDD/Si, BNDD/Ti/Si, and BNDD/Ta/Ti/Si, respectively. After detailed characterization using microscopies, spectroscopies, electrochemical techniques, and density functional theory simulations, the relationship of composition, interfacial structure, charge transport, and electrochemical properties of the interface between diamond and metal is investigated. The BNDD/Ta/Ti/Si electrodes exhibit faster electron transfer processes than the other two diamond electrodes. The interlayer thus determines the intrinsic activity and reaction kinetics. The reduction in their barrier widths can be attributed to the formation of TaC, which facilitates carrier tunneling, and simultaneously increases the concentration of electrically active defects. As a case study, the BNDD/Ta/Ti/Si electrode is further employed to assemble a redox‐electrolyte‐based supercapacitor device with enhanced performance. In summary, the study not only sheds light on the intricate relationship between interlayer composition, charge transfer, and electrochemical performance but also demonstrates the potential of tailored interlayer design to unlock new capabilities in diamond‐based electrochemical devices.

## Graphical abstract

Diamond electrochemistry is revealed to be affected by the interlayers between boron/nitrogen co‐doped nanocrystalline diamond (BNDD) film and a Si substrate. A BNDD/Ta/Ti/Si electrode exhibits faster electron transfer processes and smaller electron transfer resistance of redox probes for [Fe(CN)_6_]^3−/4−^ and [Ru(NH_3_)_6_]^3+/2+^ than the other electrodes, because the interlayer thus determines the intrinsic activity and reaction kinetics of diamond films.

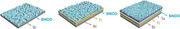

## 1 Introduction

Doped diamond, a well‐known electrode candidate in the field of electrochemistry,^[^ ^1^ ^]^ has been widely employed for electrochemical sensors,^[^ ^2^, ^3^ ^]^ supercapacitors,^[^ ^4^, ^5^ ^]^ electrocatalytic corrosion,^[^ ^6^ ^]^ electrocatalysis (e.g., oxygen reduction reaction (ORR)),^[^ ^7^ ^]^ and other applications. This is due to the unique properties of doped diamond electrodes, such as low background currents in both aqueous and non‐aqueous solutions, wide potential windows in different media, and long‐term durability in harsh environments and under extreme conditions.^[^ ^8^ ^]^ The electrochemistry of doped diamond strongly depends on the amount of sp^3^‐carbon and carbon impurities, surface terminations, and defects in diamond layers.^[^ ^9^, ^10^, ^11^, ^12^ ^]^ For example, a *p*‐type boron‐doped diamond exhibits a metal‐like conductivity once the boron doping level is above 10^20^ cm^−3^. An *n*‐type nitrogen‐doped diamond has a wide potential window to a BDD electrode.^[^ ^9^ ^]^ In recent reports, the electrochemistry of bi‐element incorporated diamond films is investigated and discussed.^[^ ^13^, ^14^, ^15^, ^16^, ^17^ ^]^ Compared with single‐dopant doped diamond, bi‐element incorporated diamond regulates the electronic structures of a diamond film, resulting in faster electron transfer rates and higher catalytic ability toward different reactions.^[^ ^18^, ^19^, ^20^ ^]^ For example, the boron and nitrogen B,N‐co‐doped diamond (BNDD) films were utilized for the ensemble of supercapacitors^[^ ^13^ ^]^ and for efficient CO_2_ reduction.^[^ ^14^ ^]^ BNDD films with adjustable nitrogen and boron dopant ratios exhibit similar performance toward the ORR as a commercial Pt/C catalyst, including high current densities for the ORR and its long‐term durability during the ORR.^[^ ^15^ ^]^

A mechanical robust substrate is required to achieve high electrochemical performance of doped diamond electrodes. Diamond films are usually grown on silicon,^[^ ^21^, ^22^ ^]^ although it is fragile and has poor mechanical properties.^[^ ^23^ ^]^ To overcome the drawbacks of silicon, other substrates (e.g., metal, carbon fibers, carbon nanotubes) were used for doped diamond electrodes.^[^ ^24^, ^25^, ^26^ ^]^ In these, as‐grown diamond films possesses low contact resistivity (using ohmic/nonrectifying contacts), good adhesion, high thermal stability, and high corrosion resistances. Taking the titanium as a promising substrate, A BDD electrode grown with Ti as the substrate has a maximum capacitance of 53.3 mF·cm^−2^ and a minimum resistance value of *R*_ct_ of 4.8 Ω. Furthermore, under certain conditions, electrical conductivity and mechanical flexibility of as‐grown diamond films were even improved.^[^ ^27^, ^28^ ^]^ Recently, other metals such as other metals (e.g., Ta, Nb) or semi‐metallic (e.g., Ge), exhibit better electrochemical performance than a Ti substrate.^[^ ^22^, ^29^, ^30^ ^]^ Surprisingly, neither the growth of diamond films on these interlayers nor the electrochemistry of diamond electrodes has been investigated thoroughly, probably due to the fact that diamond films peel off from these metal interlayers easily.^[^ ^31^ ^]^

In this study, we have grown BNDD by chemical vapor depositioin (CVD) on Ti and Ti/Ta interlayers that were sputtered on Si substrates (Figure S1, Supporting Information). The characterizations of these as‐grown diamond films with scanning electron microscope (SEM), Raman spectroscopy, secondary ion mass spectrometry (SIMS), X‐ray photoelectron spectroscopy (XPS), electron energy loss spectroscopy (EELS) confirmed the presence of C,O‐doped TiN and TaC within the interlayer. The electrochemical properties of these diamond films were systematically evaluated in both [Fe(CN)_6_]^3−/4−^ or [Ru(NH_3_)_6_]^3+/2+^ redox systems, including their potential difference of oxidation and reduction peaks, electrochemical effective active areas, double layer capacitances (*C*_dl_), potentials toward oxygen evolution reaction and hydrogen evolution reaction. To explain these electrochemical results, density functional theory (DFT) calculations were done to determine the density of states at different interlayers between Si and diamond. To further explore the electrochemical properties of these diamond films, a diamond supercapacitor was constructed, and its performance was studied.

## 2 Results and Discussion

### 2.1 Characterization of the BNDD Films

**Figure 1:**
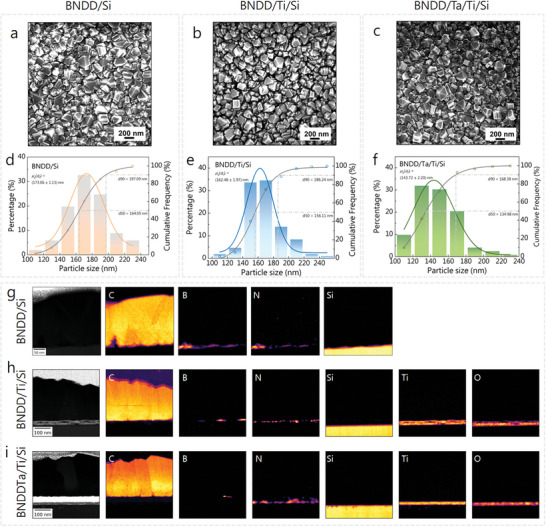
SEM images a–c), grain size statistics d–f) and STEM‐EELS elemental mapping g–i) of the BNDD/Si, BNDD/Ti/Si, and BNDD/Ta/Ti/Si, respectively, the white dashed line in (i) represents the Ta region that cannot be detected by EELS.

The SEM images of the BNDD/Si, BNDD/Ti/Si, and BNDD/Ta/Ti/Si were recorded (**Figure** **1**a–c). These films exhibit the typical morphology of polycrystalline diamond films. The surface crystal grains are clear and dense. Their particle sizes were calculated from the statistical results of particle size distribution. Specifically, for the BNDD/Si, BNDD/Ti/Si, and BNDD/Ta/Ti/Si, the respective sizes are 173, 162, and 144 nm (Figure 1d–f). Consequently, it can be inferred that the different interlayers may affect the morphologies of the particle sizes of the BNDD films. The scanning transmission electron microscope‐electron energy loss spectroscopy (STEM‐EELS) elemental mapping of C, Si, and Ti together with the simultaneously acquired high angle annular dark field (HAADF) images were also recorded (Figure 1g–i), where different interlayers are clearly shown. The diamond layer has a thickness of ≈180 nm, while the Ti and Ta sputter layers are ≈30 and 27 nm in their thickness, respectively. At the interface of the Si substrate and nanodiamond crystals, as well as within defect regions at grain boundaries, the co‐presence of B and N dopants as BN is identified based on the strong π^*^ peak of hexagonal boron nitride^[^ ^32^ ^]^ or graphitic BC_2_N^[^ ^33^ ^]^ with EELS fine structure exhibiting nearly indistinguishable characteristics^[^ ^34^ ^]^ (Figure 1g). When a titanium buffer layer is sputtered on the Si substrate, the presence of a similar BN layer at the interface between Ti and BNDD is also present. A separation within the Ti layer between a bottom O‐rich layer close to the Si‐substrate is probably related to the presence of a SiO*_x_* oxidized layer on the substrate surface and an N‐rich layer above (Figure 1h). Finally, for the bottom panels of the BNDD/Ta/Ti/Si, the BN layer is only present in asperities of the growing film and the N seems to be segregating at the interface between Ta and Ti layers (Figure 1i). Consequently, an almost continuous BN layer present is clearly identified at the interface between the BNDD and the Si substrate, as well as between the BNDD and Ti layer, but it is nearly absent for the TaC layer (Figure S2, Supporting Information).

**Figure 2:**
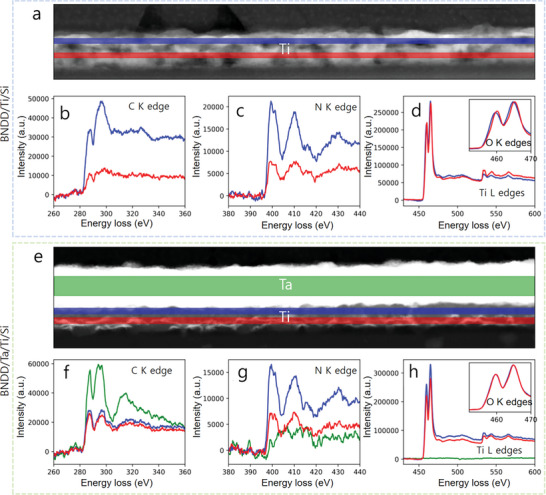
ELNES analysis of the a) BNDD/Ti/Si and e) BNDD/Ta/Ti/Si interfaces. HAADF image of the interface of the BNND/Ti/Si together with the b) C K edge, c) N K edge and d) Ti L and O K edges for two distinct regions close to the Si‐substrate in red and close the BNDD in blue. HAADF image of the interfacial region of the e) BNDD/Ta/Ti/Si with the fine structures of the f) C K edge, g) N K edge and h) Ti L and O K edges extracted the Ta layer in green, and the same two blue and red regions.

Energy‐loss near edge structure (ELNES) analysis was further performed to provide a clearer illustration of the elemental distribution in the diamond films at the interfaces of BNDD/Ti/Si and BNDD/Ta/Ti/Si (**Figure** **2**). The region of the interface between the BNND/Ti/Si is represented in Figure 2a, along with the C K edge, N K edge, and Ti L and O K edges (Figure 2b–d) for two distinct regions close to the Si‐substrate in red and close the BNND in blue. A clear shift of the Ti L edge between the two regions as well as a higher O content of the region close to the Si substrate while the blue region is richer in C and N are noticed. The fine structure of the N K edge in the Ti layers is a clear indication of the presence of TiN‐type bonding.^[^ ^35^ ^]^ The shift to higher energy observed when getting close to the Si substrate is correlated with the increase in oxygen content of the C,O‐containing TiN layer.^[^ ^36^ ^]^ On the other hand, for the interfaces BNDD/Ta/Ti/Si (Figure 2e), the fine structures of the C K edge, N K edge, and Ti L and O K edges (Figure 2f–h) are extracted for the same two blue and red regions as well as the Ta layer in green. The shift previously observed in the Ti L edge between the blue and red regions is now absent and their carbon and oxygen content became similar while the N content of the Ti layer close to the Ta layer remains very high. The carbon fine structure in the green Ta region is a clear indication of the presence of TaC, in other words, the TaC layer is clearly identified in the BNDD/Ta/Ti/Si compound.^[^ ^35^ ^]^

The Raman spectra of these BNDD films were also recorded (Figure S3, Supporting Information). The Raman peak of diamond is usually at 1332 cm^−1^. Our BNDD films show characteristic peak shifted to higher wavenumber, which is due to nitrogen and boron doping.^[^ ^36^, ^37^ ^]^ In addition, these Raman spectra have broad peaks in the range of 1500–1600 cm^−1^. It is known that the specific location of this G‐band and its intensity is determined by the relative content of carbon in the sp^2^ state in the diamond film. The sensitivity of detecting sp^2^ carbon is generally 50 times that of sp^3^ diamond using the 532 nm laser. The reduction or disappearance of the Lorentzian peak around 1200 cm^−1^ may be attributed to the incorporation of N resulting in a weakening of the Fano resonance of boron.^[^ ^38^ ^]^ The surface analysis of these diamond films was performed using XPS, focusing on the high‐resolution C1s spectra (Figure S4, Supporting Information). In these spectra, the binding energies of diamond sp^3^ carbon peaks are centered at 285.2, 285.3, and 285.3 eV for BNDD/Si, BNDD/Ti/Si, and BNDD/Ta/Ti/Si, respectively. The peaks located at 283.3, 283.8, and 283.6 eV are attributed to sp^2^ carbon, and those at 286.3, 286.8, and 286.3 eV are assigned to carbon bonded to oxygen (C−O).^[^ ^39^, ^40^ ^]^ Note that the BNDD with different interlayers exhibits no effect on the ratio of sp^2^/sp^3^ carbon (Table S1, Supporting Information). Such the sp^2^ content in XPS C1s spectra may cause by arising from the in‐plane stretching modes at the grain boundaries, while the dominant content in the bulk phase remains as sp^3^ carbon. To further illustrate the carbon sp^2^/sp^3^ hybridization of the cross‐section of these films using ELNES analysis recordings Figure S5 (Supporting Information). For each sample recorded by three regions: on top (red), center (blue), and bottom (green), with the center region clearly showing sp^2^ and sp^3^ content. Additionally, the ratio between the gray (sp^2^) and yellow (sp^3^) regions is indicated on the spectrum as a measure of sp^2^/sp^3^ hybridization. Revealing that sp^2^/sp^3^ hybridization is minimal in the bulk material, indicating that it consists predominantly of diamond. Furthermore, all of the detected boron element (re‐bonding as B^+^, ^10^B^+^, CH_3_NB^+^, CH_3_OB^+^) and nitrogen element (re‐bonding as re‐bonding as N^+^, N_2_^+^, N_2_H_3_^+^, CH_3_N^+^, C_3_H_9_N_2_^+^, CH_3_NB^+^, C_7_H_19_N_2_O^+^) are found to be homogeneously and uniformly distributed throughout the diamond surface, as confirmed from SIMS with a 3D render overlay version in these three films (Figure S6, Supporting Information).

### 2.2 Electrochemical Properties of the BNDD Films

**Figure 3:**
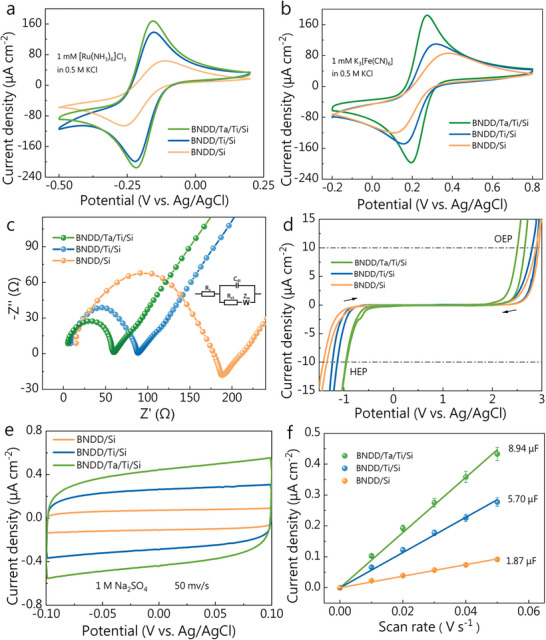
CVs of a) 1 mm [Ru(NH_3_)_6_]Cl_3_ and b) 1 mm K_3_[Fe(CN)_6_] in 0.5 m KCl on three BNDD films at a scan rate of 100 mV s^−1^. c) Nyquist plots of 1 mm K_3_[Fe(CN)_6_] in 0.5 m KCl on the BNDD films and the fitting of the equivalent circuit (inset). d) The OEP and HEP of the BNDD/Si, BNDD/Ti/Si, and BNDD/Ta/Ti/Si electrodes in 0.5 m H_2_SO_4_ solution at a scan rate of 100 mV s^−1^ e) CVs of three electrodes at a scan rate of 50 mV s^−1^ in 1 m Na_2_SO_4_, f) The variation of the current densities at 0.00 V with the applied scan rates, the error bars represent the standard deviation of three measurements of each sample (*n* = 3).

To check the electrochemical performance of these BNDD films grown on the different interlayers, they were investigated using both outer (**Figure** **3**a) and inner (Figure 3b) redox probes. In these cyclic voltammograms (CVs), a pair of well‐defined redox peaks are clearly observed. However, their peak currents and peak potentials are different on these BNDD films. In both [Ru(NH_3_)_6_]Cl_3_ and K_3_[Fe(CN)_6_] redox systems, the redox peak currents follow the order of BNDD/Si < BNDD/Ti/Si < BNDD/Ta/Ti/Si. On the BNDD/Ta/Ti/Si film, the difference in the anodic peak potential from the cathodic one is the smallest. Consequently, the BNDD/Ta/Ti/Si electrode has better electrochemical activity, toward both outer‐ and inner‐sphere redox probes. To further examine electrode kinetics of such redox probes on these diamond films, their CVs of K_3_Fe(CN)_6_ (Figure S7a–c, Supporting Information) were further recorded at different scan rates. The anodic peak currents are proportionally enhanced as a function of the square roots of scan rates (Figure S7d, Supporting Information), confirming a reversible and diffusion‐controlled electrode process on all these electrodes. According to the Randles–Sevčik equation and the slopes, the electrochemically active areas of the BNDD/Si, BNDD/Ti/Si and BNDD/Ta/Ti/Si film electrodes were calculated, which are 0.19, 0.34, and 0.85 cm^2^, respectively. These film electrodes thus have different electrochemically different active areas participate in the electrochemical reaction, apparently depending on the used interlayers.

The ohmic resistances of three diamond films were estimated by use of electrochemical impedance spectroscopy (EIS). The Nyquist plots recorded in 1 mM K_3_Fe(CN)_3_ + 0.5 m KCl solution (Figure 3c) were further fitted using the Randles equivalent electric circuit (inset in Figure 3c), where *C*_dl_ is an electric double layer capacitance, *Z*_w_ is a mass transfer impedance and related to the diffusion rate of the redox material, and *R*_s_ is the internal resistance. The value of *R*_s_ is determined by the intrinsic resistance of the active material, the ionic resistance of the electrolyte, and the contact resistance at the electroactive material/current collector interface. The calculated intrinsic resistance of BNDD/Si, BNDD/Ti/Si/, and BNDD/Ta/Ti/Si are 14.7, 8.4, and 5.3 Ω, respectively. This indicates the intrinsic conductivities of these films are dependent on the interlayers that were applied during diamond growth. The variation tendency of such a value was further co‐related with peak currents of redox probes on these films. Meanwhile, the charge transfer resistances (*R*_ct_) of these electrodes were calculated. For the BNDD/Si, BNDD/Ti/Si/, and BNDD/Ta/Ti/Si, they are 212, 145, and 59 Ω, respectively. Namely, the BNDD/Ta/Ti/Si film has the highest intrinsic conductivity and the fastest electron transfer rate.^[^ ^41^ ^]^

The electrochemical potential windows of these diamond films were evaluated. This window was defined by the potential difference in the voltammograms when the current density reached an absolution value of 10 mA cm^−2^ at a scan rate of 100 mV s^−1^. The potential at the negative potential range was named as the hydrogen evolution potential (HEP) and at the positive potential range as the oxygen evolution potential (OEP). In 0.5 m H_2_SO_4_ solution (Figure 3d), the OEPs of the BNDD/Si, BNDD/Ti/Si, and BNDD/Ta/Ti/Si electrodes are 2.88, 2.80, and 2.50 V (versus Ag/AgCl), respectively. Their HEPs are −1.35, −1.14, and −0.95 V (versus Ag/AgCl), respectively. The BNDD/Ta/Ti/Si electrode does have lower potentials toward both hydrogen evolution and oxygen evolution.

The electrochemical active surface area (ECSA) was then proceeded based on the double‐layer capacitance (*C*_dl_). The CVs of these three electrodes were further recorded within a non‐Faraday domain, namely from −0.1 to +0.1 V (Figure 3e). From these CVs recorded different scan rates in 1 m Na_2_SO_4_ solution (Figure S8, Supporting Information), the electric double layer capacitances of these electrodes were estimated by plotting out the variation of the current densities as a function of scan rates (Figure 3f). The calculated *C*_dl_ of the BNDD/Si, BNDD/Ti/Si, and BNDD/Ta/Ti/Si electrodes are 1.87, 5.70, and 8.94 µF cm^−2^, respectively.

**Figure 4:**
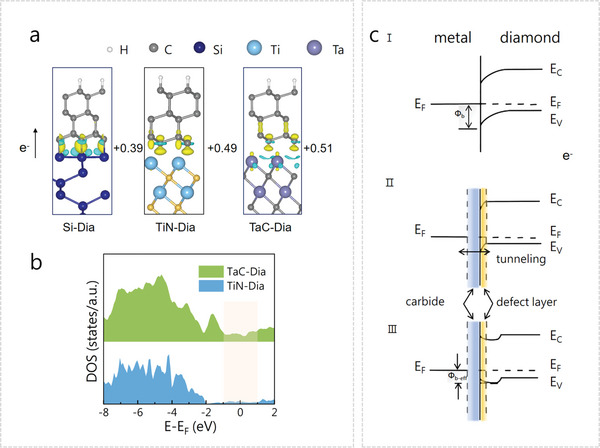
a) Charge density differences of BNDD/Si, BNDD/TiN, and BNDD/TaC interface. b) Comparison of the DOS for BNDD/TiN and BNDD/TaC interface. c) Interface band diagrams of metal layer on diamond.

Among them, the BNDD/Ta/Ti/Si electrodes exhibited faster electron transfer processes, smaller electron transfer resistance of redox probes for [Fe(CN)_6_]^3−/4−^ and [Ru(NH_3_)_6_]^3+/2+^, larger electrochemically effective active area, and more potential electrocatalytic activity than the other two diamond electrodes. The interlayer determines the intrinsic activity and reaction kinetics of diamond films, thus enhancing the electrochemical performance of these electrodes. Such the enhanced electrochemical performance of the electrodes was further studied using theoretical simulation of its interface band structure. It is known that a C,O‐doped TiN is present as an interlayer for BNDD/Ti/Si, while the presence of a TaC interlayer for BNDD/Ta/Ti/Si is confirmed. The DFT calculations were thus conducted to investigate the influence of different interlayers on the interface band structures of three diamond films. For these simulations, six‐layers of diamond slabs with ideal (111) surfaces were extracted from the bulk diamond structure. The number of layers applied was found to be large enough to capture the change in the electronic structure from the surface to its bulk.^[^ ^42^ ^]^ For BNDD/Si, BNDD/TiN, and BNDD/TaC electrodes, several models of monolayers were constructed (Figure S9, Supporting Information), the difference of the Bader charges and charge densities on the interfaces were then calculated (**Figure** **4**a). From the calculated electron density of states (DOS), one can see that the charge transfer is induced from the metal atoms to the BNDD. The charge densities at the BNDD/TaC interface are stronger than those at both BNDD/TiN and BNDD/Si interfaces, namely with a value shift from +0.39 to +0.51 eV^−1^. To further investigate the influence of metal nitride/carbides, the DOS of the BNDD/TiN and BNDD/TaC interfaces were calculated and compared (Figure 4b). The DOS near the Fermi level appears to exhibit an increase between TaC and the diamond layer, where a more pronounced effect is observed when TaC is present. This enhanced charge transfer significantly impacts the electronic structures of bonding atoms. An increase in the density of states means more charge transfer at the interface, indicating better electrical contact. Furthermore, the metal carbides from tantalum have been utilized to achieve an ohmic contact on the diamond surface.^[^ ^43^ ^]^ The formation of such an ohmic contact was speculated by the defect generation at and near the diamond surface via the carbide‐forming reaction. The presence of such defects either narrows the depletion width, thereby increasing the probability of tunneling, or reducing the effective barrier height (Figure 4c).^[^ ^34^ ^]^ In other words, the presence of metal carbides is mainly responsible for the difference in the electrochemical properties of as‐grown diamond films.

### 2.3 Electrochemical Applications of the BNDD Films

**Figure 5:**
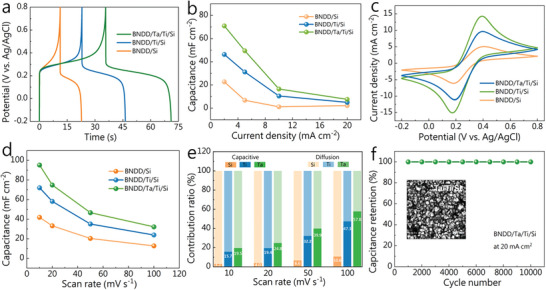
Capacitive performance of the BNDD electrodes in 0.05 M Fe(CN)_6_^3−/4−^+ 1.0 M Na_2_SO_4_: a) Charge/discharge curves of the BNDD/Si, BNDD/Ti/Si, and BNDD/Ta/Ti/Si electrodes at a current density of 2 mA cm^−2^. b) The variation of the specific capacitances with current densities. c) CVs recorded on the BNDD/Si, BNDD/Ti/Si, and BNDD/Ta/Ti/Si electrodes at a scan rate of 10 mV s^−1^. d) The variation of the specific capacitances with scan rates. e) The contribution ratios of capacitive and diffusion capacities as a function of scan rates; f) The capacitance retention of the BNDD/Ta/Ti/Si electrode at a current density of 20 mA cm^−2^ after 10 000 cycles. The inset is a SEM image of the BNDD/Ta/Ti/Si electrode after 10 000 charge/discharge cycles at a current density of 20 mA cm^−2^.

The electrochemical applications of such diamond films have been explored. Compared to electrical‐double‐layer capacitors (EDLCs), pseudocapacitors (PCs) rely on surface‐controlled faradaic reactions of redox‐active materials at the electrode/electrolyte interface. The additional charge transfer resulting from these redox reactions leads to higher capacitances in pseudocapacitors. Taking the capacitance of a pseudocapacitor using Fe(CN)_6_^3‐/4−^ redox couples as an example, it is about fourorder higher than that of a diamond EDLC.^[^ ^5^, ^12^ ^]^ As a case study, their potential of being employed for the construction of a supercapacitor has been evaluated. In a three‐electrode system, the galvanostatic charge/discharge (GCD) curves of these BNDD electrodes were recorded at different current densities (**Figure** **5**a). These electrodes have good reversibility, as evidenced by the nearly equal charge and discharge times in these GCD curves. The calculated specific capacitances for the BNDD/Si, BNDD/Ti/Si, and BNDD/Ta/Ti/Si electrodes at a current density of 2 mA cm^−2^ are 22.7, 46.3, and 71.0 mF cm^−2^, respectively. From the GCD curves of the BNDD/Ta/Ti/Si electrodes recorded at different current densities (Figure S10, Supporting Information), its calculated capacitances are 71.0, 49.5, 16.5, and 7.6 mF cm^−2^, respectively (Figure 5b). They are higher than the BNDD/Ti/Si electrode (e.g., 46.3, 31.2, 10.4, and 5.0 mF cm^−2^, respectively) and the BNDD/Si electrode (e.g., 22.7, 17.0, 5.8, and 2.2 mF cm^−2^, respectively).

The CVs of these diamond electrodes are compared at the same redox electrolyte at a scan rate of 10 mV s^−1^ (Figure 5c). From these CVs, the capacitances of the BNDD/Ta/Ti/Si electrode were calculated. At the scan rates of 10, 20, 50, and 100 mV s^−1^, they are 95.3, 74.9, 46.6, and 32.2 mF cm^−2^, respectively (Figure 5d; Figure S11, Supporting Information), which are higher than those of the BNDD/Ti/Si electrode (72.1, 58.2, 35.1, and 23.9 mF cm^−2^, respectively) and the BNDD/Si electrode (41.9, 33.2, 20.4, and 17.3 mF cm^−2^, respectively). As control experiments, the CVs of these electrodes were recorded in 1.0 m Na_2_SO_4_ at different scan rates (Figure S12, Supporting Information). The estimated capacitances of the BNDD/Si, BNDD/Ti/Si, and BNDD/Ta/Ti/Si electrodes at a scan rate of 10 mV s^−1^ are only 53.1, 9.5, and 7.5 µF cm^−2^. Therefore, the addition of redox electrolytes into the solution enhances ≈1000 times of the capacitances of these electrodes, as expected for such redox‐electrolytes enhanced (pseudo)capacitors.^[^ ^44^, ^45^, ^46^ ^]^

For such redox‐electrolytes enhanced (pseudo)capacitors, their kinetics can be further analyzed. Namely the contributions of diffusion‐bound and surface‐bound species can be further quantitatively discretized using the equation of *i*(*v*) = *k*_1_ *v* + *k*_2_ *v* ^1/2^, where *k*_1_ *v* and *k*_2_ *v* ^1/2^ correspond to the capacitive effect and diffusive contribution, respectively.^[^ ^47^, ^48^ ^]^ The constants of *k*_1_ and *k*_2_ were then calculated based on the simplified equation of *i*(*v*)/*v* ^1/2^ = *k*_1_ *v* ^1/2^ + *k*_2_ at various scan rate (*v*) (Figure 5e). As the scan rate increases, the capacitive contribution gradually increases. Specifically, the BNDD/Ta/Ti/Si electrode shows a higher capacitive contribution than the BNDD/Si and BNDD/Ti/Si electrodes. This reveals the better potential of the BNDD/Ta/Ti/Si electrode for electrochemical energy storage applications.

The long‐term cycling stability of these BNDD electrodes was further tested using the GCD method at a current density of 20 mA cm^−2^. All these electrodes exhibit high and stable capacitances (Figure 5f; Figure S13, Supporting Information), as confirmed from the SEM images for these electrodes after long‐term tests (e.g., 10 000 GCD cycles). Further comparisons of the capacitors performance of BNDD/Ta/Ti/Si electrode with reported by using other doping diamond‐based electrode materials are summarized in Table S3 (Supporting Information). It can be concluded that such a modified co‐doped diamond electrode exhibits better capacitor performance. Therefore, all these results confirm the suitability of co‐doped diamond films grown on the metal interlayers for electrochemical energy storage applications.

## 3 Conclusion

The influence of interlayers on the electrochemistry of B,N‐co‐doped diamond films has been revealed experimentally and theoretically. The diamond film grown on a Ta/Ti/Si interlayer exhibits better electrochemical response toward inner‐spherical and outer‐spherical redox systems than those grown on a Si substrate and a Ti interlayer. The reasons behind are assumed to stem from its smaller electrochemical fitting resistance and its faster electron transfer rate. The improvement of electrochemical performance of the BNDD/Ti/Ta film is related to the smaller particle size and the presence of TaC between the Si and the diamond layer. This film is promising to be applied to the assembling of high‐performance supercapacitors. Future work can be conducted in the fields of electrocatalysis where a low potential of the hydrogen evolution reaction is required, such as for electrochemical CO_2_ reduction reaction, and electrochemical nitrogen fixation. Meanwhile, such an electrode is attractive for environmental applications where a high potential of the oxygen evolution reaction is required (e.g., pollutant degradation).

## 4 Experimental Section

### Material Synthesis

The thin metal films were deposited by reactive direct current‐pulsed magnetron sputtering on (100) oriented Si substrates. Prior to deposition, the substrates were exposed to oxygen plasma for the removal of surface contaminants. The sputtering targets were 4 inches in diameter, held on a water‐cooled magnetron cathode. The distance between the target and the substrate holder was 10 cm. Before the deposition, targets were cleaned using argon gas discharge plasma for 5 min, followed by a pre‐sputtering stage, using the same conditions as the subsequent film deposition with a shutter shielding the sample. The base pressure in the sputtering chamber was below 1 × 10^−5^ mbar, while the deposition pressure was kept at 4.5 × 10^−3^ mbar. Table S2 (Supporting Information) shows the parameters of the metal sputtering.

The BNDD films growth was carried out in an ASTeX 6500 series microwave plasma‐enhanced chemical vapor deposition (MW PE CVD) reactor. The growth temperature was monitored by a Williamson Pro92 dual‐wavelength pyrometer. The CH_4_, N_2_, H_2_, trimethyboron (TMB) were flown at a rate of 5, 10, 385, and 100 sccm, respectively (The TMB is diluted to 1000 ppm in H_2_). This gas composition gives [B]/[C] = 20 000 ppm. Under such conditions, the nitrogen concentration was kept as 2%. A substrate temperature of 700 °C was induced by a microwave power of 4000 W at a total pressure of 40 Torr. The growth time of these BNDD films was 2 h.

### Material Characterization

The SEM images of the BNDD films grown on Si, Ti, and Ta interlayers were recorded with a field emission scanning electron microscope (FESEM, Zeiss ultra55, Germany). The microstructure of the BNDD films with different interlayers were analyzed using a Thermofisher Titan ThemisZ microscope operating at 60 kV. The convergence and collection angle for STEM‐EELS was set respectively to 16 and 40 mrad. The dispersions were 170 meV pixel^−1^ and the energy spread of the incoming electrons was 700 meV. Low and core loss were acquired almost instantaneously with a dwell time of 20 ms and incoming electron current of 30 pA. Model‐based fitting procedure^[^ ^49^ ^]^ was used to extract the elemental maps where a linear background model was used^[^ ^50^ ^]^ and the core‐loss edges were modeled using the atomic cross‐sections.^[^ ^51^ ^]^ The Raman spectra of these BNDD films were measured by a homemade Raman microscope around an inverted microscope (Nikon TE300, objective lenses from Nikon and optical Filters from AHF Analysentechnik) equipped with a 532 nm laser (Coherent Compass 315M‐100, 100 mW output power) as excitation source, a monochromator (Jobin Yvon, TRIAX 320, with a 1200 l mm^−1^ grating) and a spectroscopic back‐illuminated EMCCD camera (Oxford Instruments Andor, Newton EMCCD DU970P) as detection unit. The surface analysis of these as‐grown diamond films was done using X‐ray photoelectron spectroscopy (XPS) (S‐probe ESCA SSX‐100s, Surface Science Instruments, USA) with Al Kα radiation of 200 W. The XPS spectra were collected from 0 to 1200 eV with a resolution of 1 eV and a spot size of 800 µm^2^. The high‐resolution XPS spectra were collected at 0.1 eV resolution at a spot size of 300 µm^2^. A time‐of‐flight secondary ion mass spectrometer (ToF‐SIMS IV, ION‐TOF GmbH, Germany) was used to map the dopants in these diamond films in the positive mode, such as the contents of nitrogen and boron atoms in the BNDD films. For these mapping experiments, a 25 keV Bi^+^ primary ion beam was used to bombard the diamond surface within an area of 300 × 300 µm^2^ after 1200 s sputtering. Density‐functional theory (DFT) calculations, including geometry relaxation and electronic structure calculations, were conducted on the Vienna ab initio Simulation Package (VASP) with the exchange‐correlation functions of Perdew–Burke–Ernzerhof (PBE) and generalized‐gradient approximation (GGA). The plane‐wave kinetic‐energy cutoff was set at 450 eV effect. For all models, six layers of diamond (111) were combined with several layers of metal and metal carbide. A vacuum layer of 10 Å was adopted to avoid interactions at the interface. The size of the cell surface region we established is ≈5 Å by 5 Å, which reflects the properties of a certain microscopic region of the periodic electrode structure. For geometry optimization, the Brillouin zone was settled with 3 × 3 × 1 *k* points. For electronic structure 9 × 9 × 1 *k* points were employed for optimal results. Geometry optimization was employed for all systems until the residual forces converged to 0.01 eV Å^−1^ and the energy criterion was 10^−6^ eV.

### Electrochemical Measurements

All electrochemical measurements were carried out on a CHI660E workstation (Shanghai Chenhua Company, China). A classic three‐electrodes system was applied, where the reference electrode was an Ag/AgCl electrode, the counter electrode was a Pt wire, and the working electrode was the BNDD film. The geometric area of the working electrode was 0.05 cm^2^. For the construction of diamond‐based electric double‐layer capacitors (EDLCs) and pseudocapacitors (PCs), 1.0 m Na_2_SO_4_ and 1.0 m Na_2_SO_4_ containing 0.05 m K_3_Fe(CN)_6_/K_4_Fe(CN)_6_ aqueous solutions were employed, respectively. Their performance was evaluated through cyclic voltammetry at various scan rates, galvanostatic charging/discharging method at different current densities, and electrochemical impedance spectroscopy technique at open circuit potentials within the frequency range of 0.01 to 10^6^ Hz.

## Conflict of Interest

The authors declare no conflict of interest.

## Supporting information

Supporting Information

## Data Availability

Research data are not shared.
