# Supplementary material for: Interlayer Affected Diamond Electrochemistry
Source: Small Methods. 2024 Jun 14;9(2):2301774. doi: 10.1002/smtd.202301774 (PMC11843408; doi:10.1002/smtd.202301774)
Supplement: Supplementary file 1 — Supporting Information [file SMTD-9-2301774-s001.docx]

**Supporting Information**

Interlayer affected diamond electrochemistry

Xinyue Chen, Ximan Dong, Chuyan Zhang, Meng Zhu, Essraa Ahmed, Giridharan Krishnamurthy, Rozita Rouzbahani, Paulius Pobedinskas, Nicolas Gauquelin, Daen Jannis, Kawaljit Kaur, Aly Mohamed Elsayed Hafez, Felix Thiel, Rainer Bornemann, Carsten Engelhard, Holger Schönherr, Johan Verbeeck, Ken Haenen, Xin Jiang, and Nianjun Yang*

X. Chen, X. Dong, C. Zhang, M. Zhu, X. Jiang

Institute of Materials Engineering

University of Siegen

57076 Siegen, Germany

E. Ahmed, G. Krishnamurthy, R. Rouzbahani, P. Pobedinskas, K. Haenen

Institute for Materials Research (IMO), Hasselt University

and IMOMEC, IMEC vzw

3590 Diepenbeek, Belgium

N. Gauquelin, D. Jannis, J. Verbeeck

Electron Microscopy for Materials Research (EMAT)

University of Antwerp

2020 Antwerp, Belgium

K. Kaur, H. Schönherr

Physical Chemistry I

Department of Chemistry and Biology and Department of Chemistry and Biology and Research Center of Micro and Nanochemistry and (Bio)Technology (Cμ)

University of Siegen

57075 Siegen, Germany

A. M. Hafez, C. Engelhard

Analytical Chemistry

Department of Chemistry and Biology and Research Center of Micro and Nanochemistry and (Bio)Technology (Cμ)

University of Siegen,

Siegen, 57075

F. Thiel, R. Bornemann

Institute for High Frequency and Quantum Electronics

University of Siegen

57076 Siegen, Germany

N. Yang

Department of Chemistry, Hasselt University

and IMOMEC, IMEC vzw

3590 Diepenbeek, Belgium

**Supporting Tables**

**Table S1**. Relative abundance of the carbon components in the BNDD/Si, BNDD/Ti/Si, and BNDD/Ta/Ti/Si^*^.

|  | sp^2^ C | sp^3^ C | C-O | sp^2^ C/sp^3^ C |
| --- | --- | --- | --- | --- |
| BNDD/Si | 46.38 | 31.71 | 6.11 | 1.46 |
| BNDD/Ti/Si | 48.08 | 36.72 | 10.64 | 1.31 |
| BNDD/Ta/Ti/Si | 43.75 | 36.56 | 7.74 | 1.20 |

^*^ These atomic ratios were estimated from their high resolution C1s XPS spectra.

**Table S2.** Parameters of the metal sputtering

| Parameter | Ti | Ta |
| --- | --- | --- |
| Base Pressure (10^−6^mbar) | 6.9 | 7.4 |
| Working Pressure (10^−3^mbar) | 4.5 | 4.5 |
| Power (W) | 150 | 150 |
| Voltage (V) | 152.8 | 155.1 |
| Current (A) | 0.98 | 0.97 |
| Ar Gas (sccm) | 50 | 50 |
| Time (min : s) | 01:11 | 01:08 |

**Table S3.** Comparison of performance on different diamond-based electrodes. (As measured using a three-electrode system in 0.05 M Fe(CN)_6_^3-/4^ and 1.0 M Na_2_SO_4_.)

| Electrode | Capacitance | Lifetime | Ref. |
| --- | --- | --- | --- |
| BDD | 41.51 mF cm^-2^  (10 mV s^-1^) | 100%  (6 000 cycles, 5 mA cm^-2^) | [1] |
| BDD network | 73.42 mF cm^-2^  (10 mV s^-1^) | 100%  (12 000 cycles, 5 mA cm^-2^) | [1] |
| PDD  (post-thermal treatment) | 63.56 mF cm^-2^  (20 mV s^-1^) | - | [2] |
| TiC/BDD | 42.6 mF cm^-2^  (10 mV s^-1^) | 92%  (10 000 cycles, 20 mA cm^-2^) | [3] |
| Porous BDD | 78.23 mAh g^-1^  (10 mV s^-1^) | 92.6%  (5 000 cycles, 200 mV s^-1^) | [4] |
| NBD | 87.8 mF cm^-2^  (10 mV s^-1^) | -  (Stable after 10 000 cycles) | [5] |
| Graphite@NDD nanoneedles | 66.65 mF cm^-2^  (10 mV s^-1^) | 100.0%  (10 000 cycles, 10 mA cm^-2^) | [6] |
| 3D diamond/graphite | 889 mF cm^-2^  (3 mA cm^-2^) | 118.0%  (50 000 cycles, 30 mA cm^-2^) | [7] |
| core-shell diamond-graphite hybrid  nano-needles | 190 mF cm^-2^  (3 mA cm^-2^) | 96.1%  (10 000 cycles, 3 mA cm^-2^) | [8] |
| sp^3^ and sp^2^ carbon hybrids | 400 mF cm^-2^  (10 mV s^-1^) | 95%  (10 000 cycles, 3 mA cm^-2^) | [9] |
| **BNDD/Ta/Ti/Si** | **95.3 mF cm^-2^**  **(10 mV s^-1^)** | **100%**  **(10 000 cycles, 20 mA cm^-2^)** | **This work** |

**Supporting Figures**


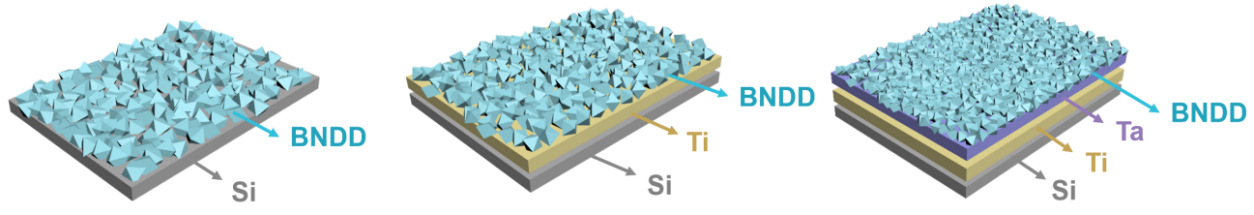


**Figure S1.** Structure of BNDD films on different interlayers.


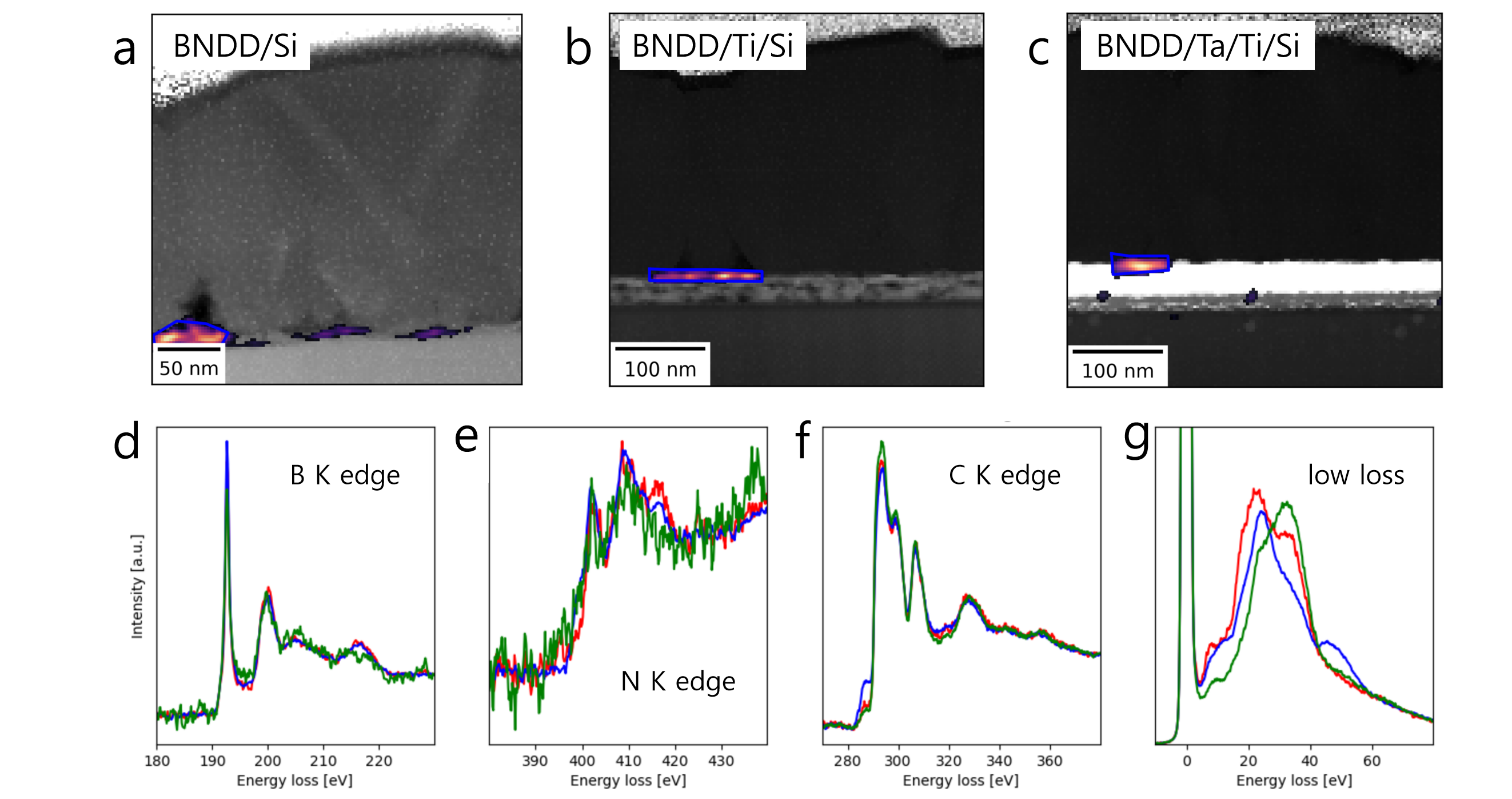


**Figure S2.** Analysis of the BN regions present at the interface between the BNDD and the coated/uncoated Si substrate. Overlay of the B-elemental map with the HAADF image for BNDD/Si (a), BNDD/Ti/Si (b), and BNDD/Ta/Ti/Si (c) the respective spectra of the B K edge (d), N K edge (e), C K edge (f) and the plasmon region (g) are reported for BNDD/Si (red), BNDD/Ti/Si (blue), and BNDD/Ta/Ti/Si (green)


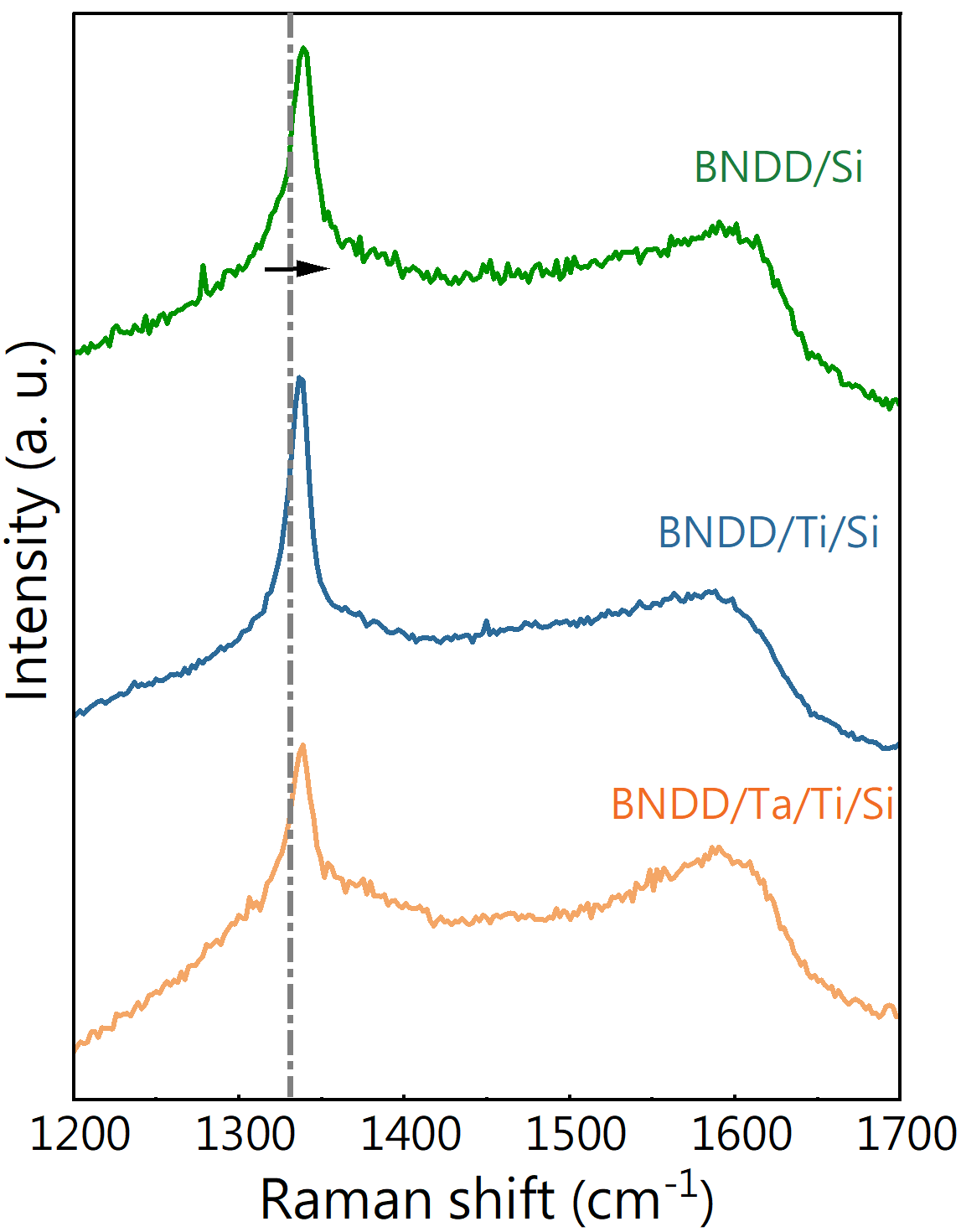


**Figure S3.** Raman spectra of BNDD/Si, BNDD/Ti/Si, and BNDD/Ta/Ti/Si.


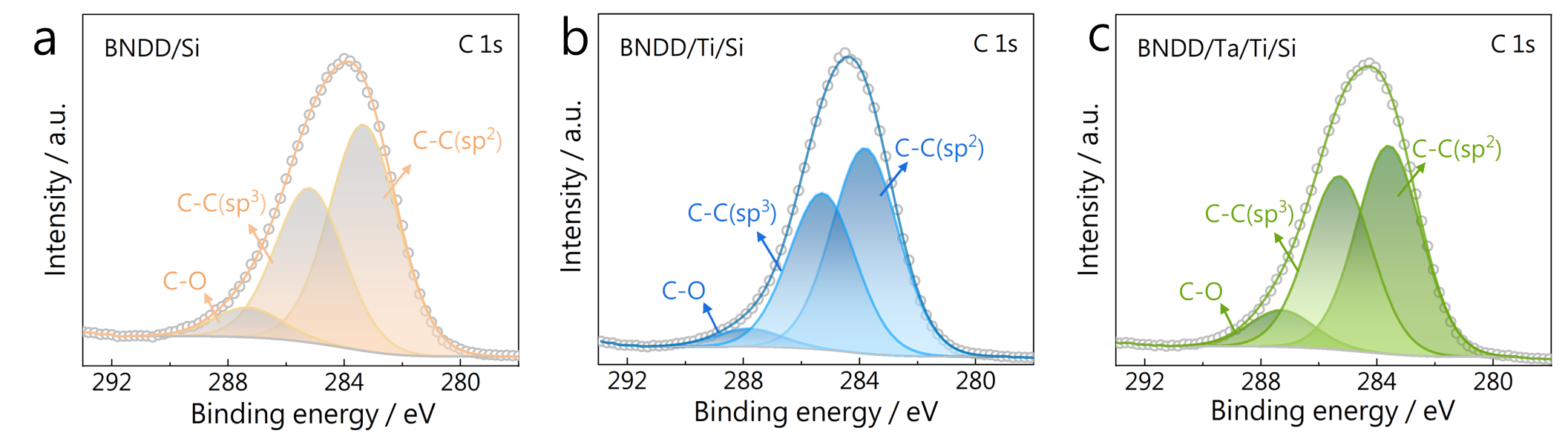


**Figure S4.** C1s XPS spectra of the (a) BNDD/Si, (b) BNDD/Ti/Si, and (c) BNDD/Ta/Ti/Si.


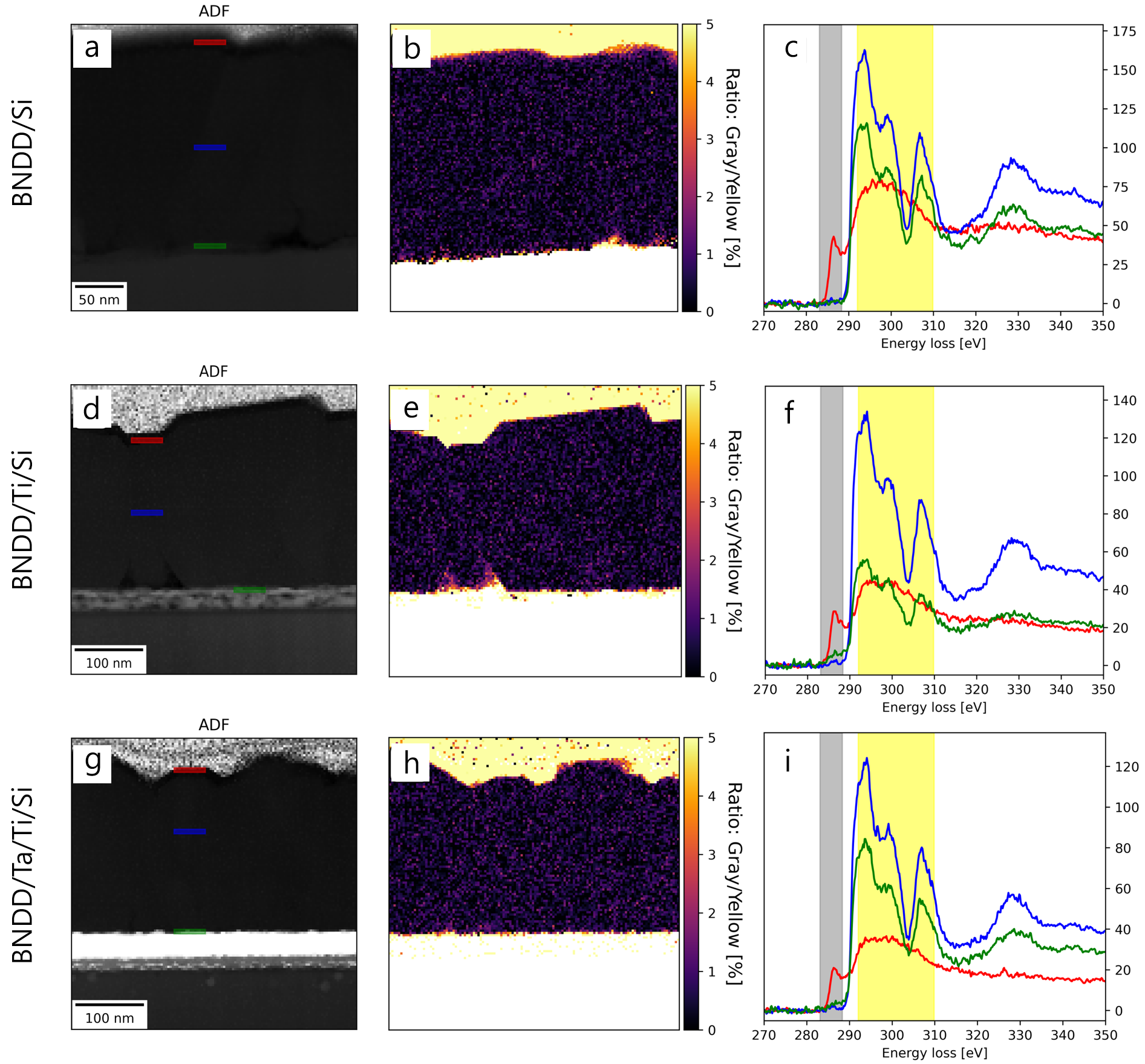


**Figure S5.** Analysis of the sp^2^/sp^3^ hybridization regions by top (red), center (blue), and bottom (green) (a, d, g). The ratio between the gray (sp^2^) and yellow (sp^3^)(b, e, h), the respective spectra of the C K edge (c, f, i) are reported for top (red), center (blue), and bottom (green) for BNDD/Si, BNDD/Ti/Si, and BNDD/Ta/Ti/Si.


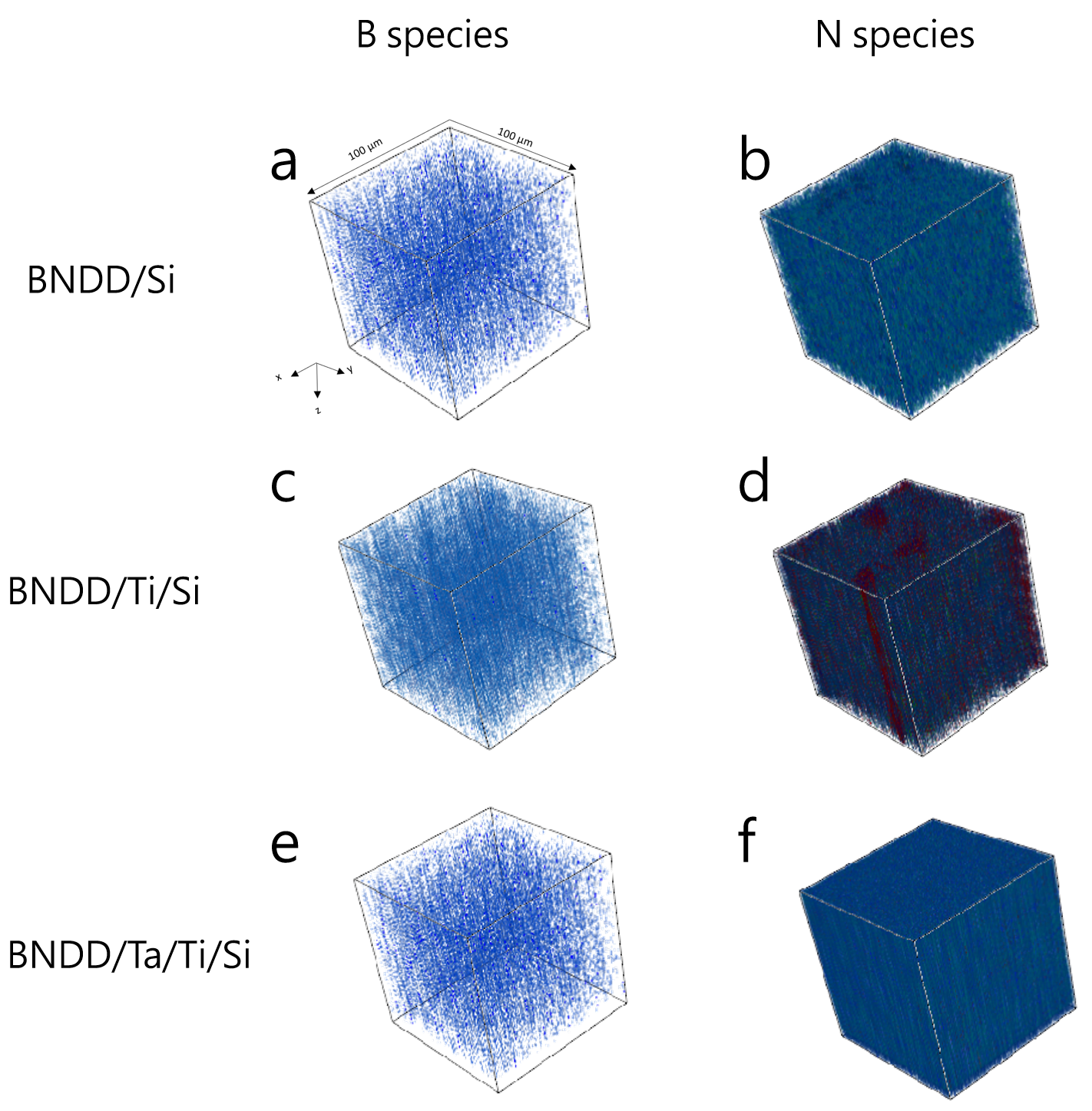


**Figure S6.** The 3D render images overlay SIMS of boron- and nitrogen-doped species in (a, b) BNDD/Si, (c, d) BNDD/Ti/Si, and (e, f) BNDD/Ta/Ti/Si. The x, y dimensions of the analyzed area are 100 μm × 100 μm, while the vertical z axis corresponds to the depth according to the sputtering time with the Bi+ primary ion beam (25 keV)


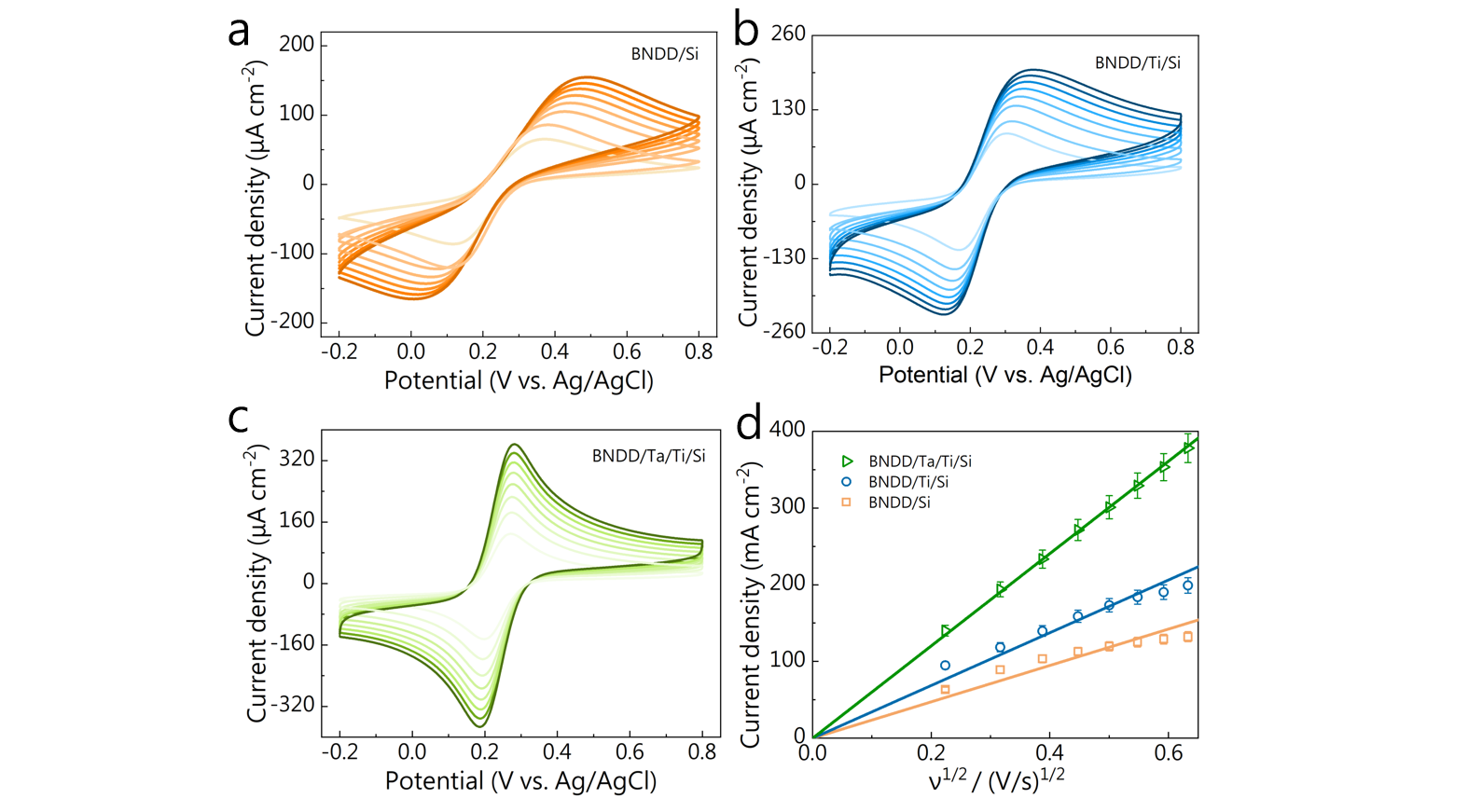


**Figure S7**. CVs of (a) the BNDD/Si, (b) BNDD/Ti/Si and (c) BNDD/Ta/Ti/Si electrodes in 1 mM K_3_Fe(CN)_6_ and 0.1 M KCl at different scan rates. The variation of anode peak currents with square roots of scan rates on these films.


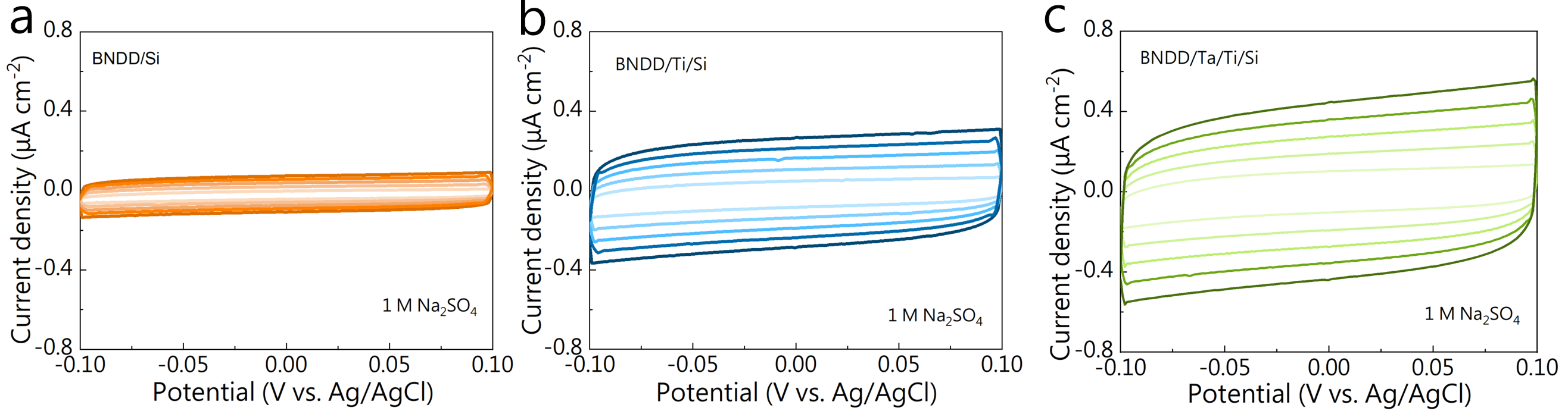


**Figure S8**. CVs of the (a) BNDD/Si, (b) BNDD/Ti/Si and (c) BNDD/Ta/Ti/Si electrodes in 1 M Na_2_SO_4_ at different scan rates.


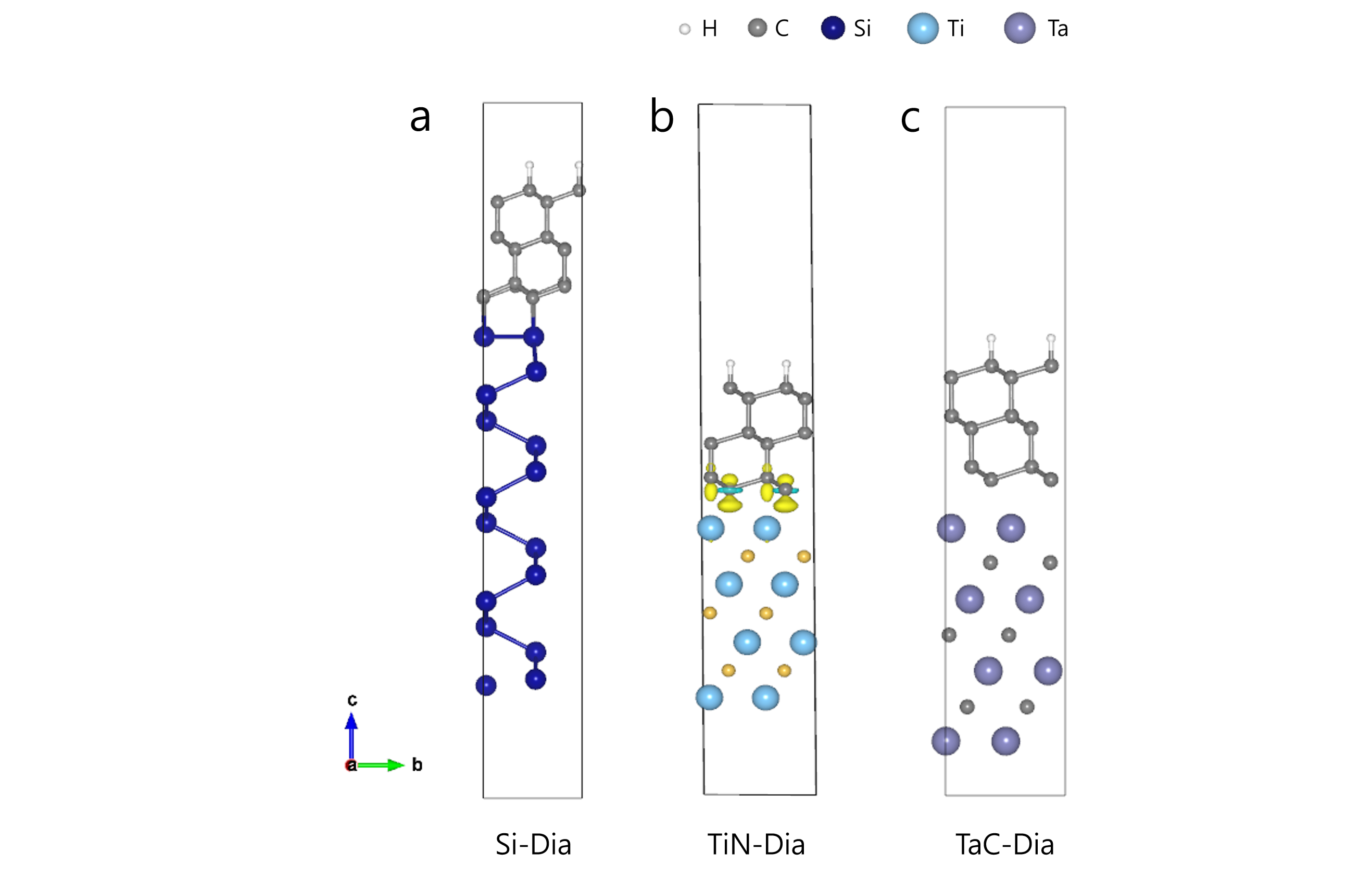


**Figure S9.** The constructed models of (a) BNDD/Si, (b) BNDD/TiN, and (c) BNDD/TaC.


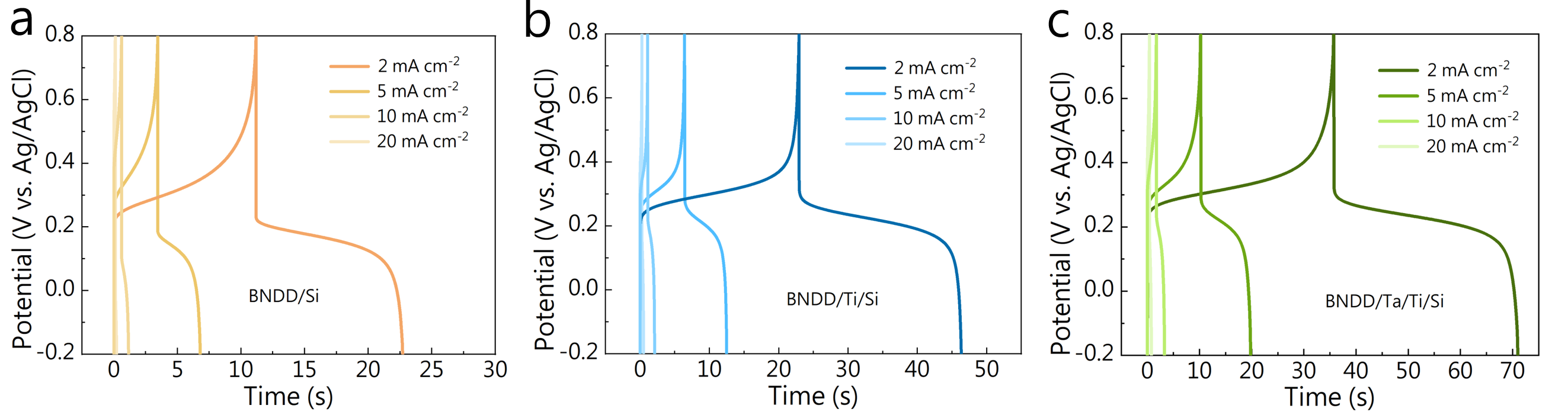


**Figure S10**. GCD curves of the (a) BNDD/Si, (b) BNDD/Ti/Si, and (c) BNDD/Ta/Ti/Si electrodes in 0.05 M Fe(CN)_6_^3−/4−^ + 1.0 M Na_2_SO_4_ at different current densities.


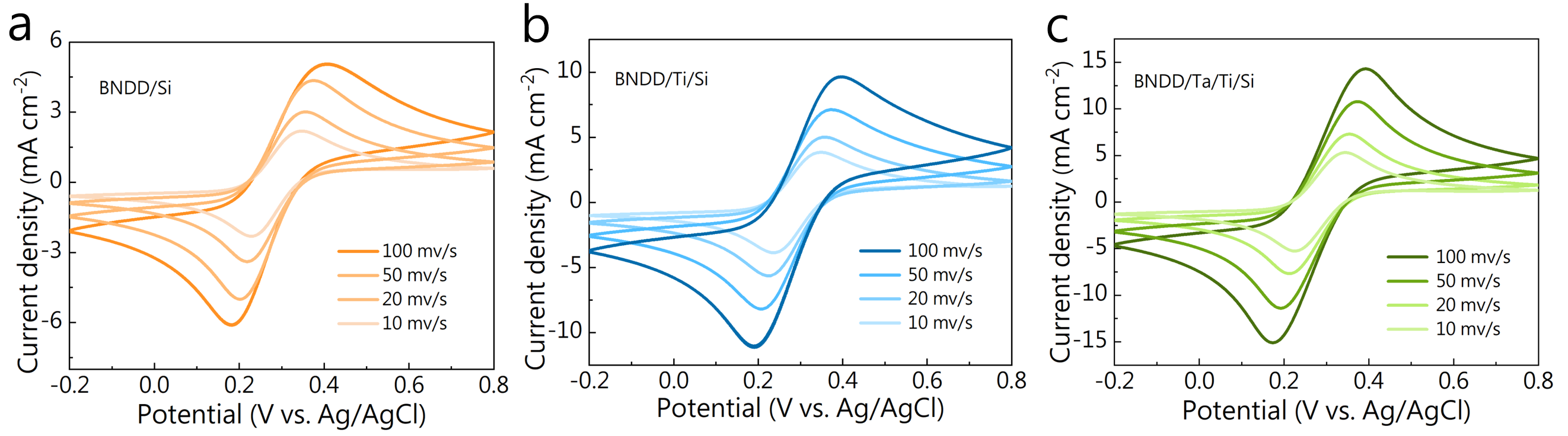
**Figure S11**. CVs of the (a) BNDD/Si, (b) BNDD/Ti/Si, and (c) BNDD/Ta/Ti/Si electrodes in 0.05 M Fe(CN)_6_^3−/4−^ + 1.0 M Na_2_SO_4_ at different scan rates.


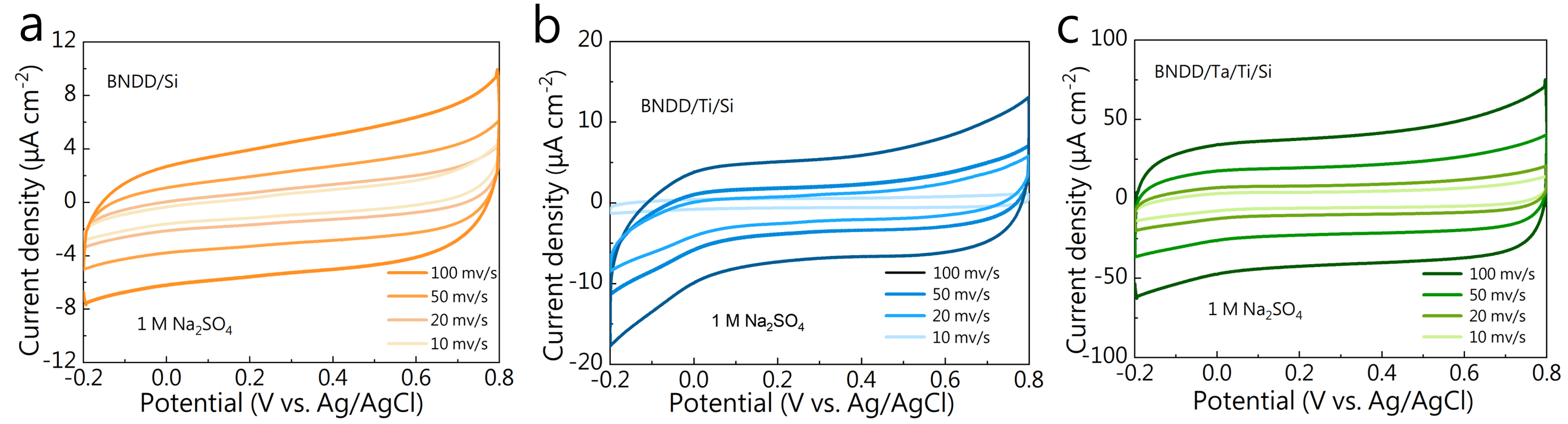


**Figure S12**. Capacitive performance of three electrodes in 1.0 M Na_2_SO_4_: (a) CVs of the BNDD/Si, BNDD/Ti/Si, and BNDD/Ta/Ti/Si electrode at a scan rate of 100 mV s^−1^; (b) the variation of the specific capacitances with scan rates; CVs of the (c) BNDD/Si, and (d) BNDD/Ti/Si and (e) BNDD/Ta/Ti/Si electrodes at different scan rates.


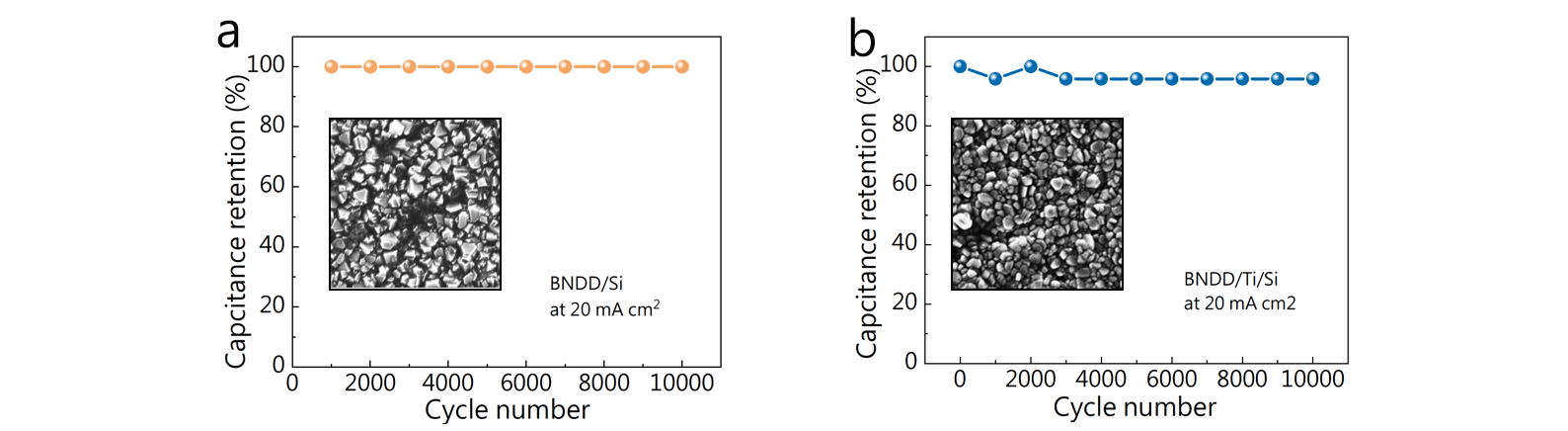


**Figure S13**. The capacitance retention of the (a) BNDD/Si and (b) BNDD/Ti/Si electrodes at a current density of 20 mA cm^-2^ after 10000 GCD cycles. The insets show the SEM images of the (a) BNDD/Si and (b) BNDD/Ti/Si electrode after 10000 GCD cycles.

**References**

[1] S. Yu, N. Yang, H. Zhuang, S. Mandal, O.A. Williams, B. Yang, N. Huang, X. Jiang, Battery-like supercapacitors from diamond networks and water-soluble redox electrolytes. *J. Mater. Chem. A*, **2017**, *5*, 1778–1785.

[2] S. Yu, J. Xu, H. Kato, N. Yang, A. Schulte, H. Schönherr, X. Jiang, Phosphorus‐doped nanocrystalline diamond for supercapacitor application, *ChemElectroChem* **2019**, *6*, 1088–1093.

[3] J. Xu, N. Yang, S. Heuser, S. Yu, A. Schulte, H. Schönherr, X. Jiang, Achieving ultrahigh energy densities of supercapacitors with porous titanium carbide/borondoped diamond composite electrodes, *Adv. Energy Mater*. **2019**, *9,* 1803623.

[4] J. Wang, Z. He, X. Tan, T. Wang, X. He, L. Zhang, J. Huang, G. Chen, K. Du, Hybrid supercapacitors from porous boron-doped diamond with water-soluble redox electrolyte, *Surf. Coat. Technol.* **2020**, *398*, 126103.

[5] T. Guo, N. Yang, B. Yang, A. Schulte, Q. Jin, U. Koch, S. Mandal, C. Engelhard, O.A. Williams, H. Schönherr, X. Jiang, Electrochemistry of nitrogen and boron bi-element incorporated diamond films, *Carbon* **2021**, *178*, 19–25.

[6] S. Yu, K.J. Sankaran, S. Korneychuk, J. Verbeeck, K. Haenen, X. Jiang, N. Yang, High-performance supercabatteries using graphite@diamond nano-needle capacitor electrodes and redox electrolytes, *Nanoscale* **2019**, *11*, 17939–17946.

[7] J. Long, L. Guan, J. Wang, H. Liu, B. Wang, Y. Xiong, Battery-like flexible supercapacitors from vertical 3D diamond/graphite composite films on carbon cloth, *Carbon* **2022***, 197*, 400–407

[8] D. Banerjeea, K.J. Sankaranb, S. Deshmukha, C.J. Yehc, M. Gupta, I-N. Lin, K. Haenene, A. Kanjilal, S.S. Roya, Single-step synthesis of core-shell diamond-graphite hybrid nano-needles as efficient supercapacitor electrode, *Electrochim. Acta.* **2021**, *397*, 139267.

[9] D. Banerjee, K.J. Sankaran, S. Deshmukh, M. Ficek, C.J. Yeh, J. Ryl, I-N. Lin, R. Bogdanowicz, A. Kanjilal, K. Haenen, S.S. Roy, Single-step grown boron doped nanocrystalline diamond-carbon nanograss hybrid as an efficient supercapacitor electrode, *Nanoscale*, **2020**, *12*, 10117.
